# A Rapid Fenton treatment of bio-treated dyeing and finishing wastewater at second-scale intervals: kinetics by stopped-flow technique and application in a full-scale plant

**DOI:** 10.1038/s41598-019-45948-9

**Published:** 2019-07-04

**Authors:** Yunlu Chen, Yunqin Cheng, Xiaohong Guan, Yan Liu, Jianxin Nie, Chenxi Li

**Affiliations:** 1 Department of Environmental Science and Engineering, Fudan University, Shanghai, 200433 China; 2 College of Environmental Science and Engineering, Tongji University, Shanghai, 200092 China

**Keywords:** Pollution remediation, Environmental chemistry

## Abstract

A rapid Fenton treatment at second-scale intervals was investigated for further removal of organic compounds in the effluent of bio-treated dyeing and finishing wastewater (BDFW). The decolorization kinetics was studied using a stopped-flow spectrophotometer (SFS) at second-scale intervals. A combined first-order model was found to fit well for the decrease of both methylene blue and rhodamine B in SFS as well as SCOD (soluble chemical oxygen demand) and DOC (dissolved organic carbon) in real BDFW in batch test during Fenton oxidation. A full-scale plant with treatment capacity of 400,000 m^3^·d^−1^ was designed and has been run continuously based on the results of the stopped-flow study to treat the effluent of BDFW using Fenton oxidation in 16 pipeline reactors, each with a volume of 6.9 m^3^ and 24 s of reaction time since 2014. The COD, SCOD and DOC decreased from 140, 110 and 35 mg·L^−1^ to 77, 71 and 26 mg·L^−1^ respectively, which can meet the latest strict discharge limitations. The natural fluorescent substances detected in the BDFW were completely removed. The main organic pollutants in the BDFW can be significantly reduced using both gas chromatography-mass spectrometry and ultrahigh-performance liquid chromatography-hybrid quadrupole time-of-flight mass spectrometry. The rapid Fenton reaction applied in pipeline reactors at second intervals has several advantages over the conventional Fenton’s process such as much shorter reaction time at second scale intervals, no need to build extra pH adjustment or reaction tanks, simple operation, low capital cost, etc.

## Introduction

With the rapid development of textile dyeing and finishing industry, water pollution due to refractory organic pollutants has become a serious issue. Such organic pollutants that are characterized by high levels of chemical oxygen demand (COD), colour and toxicity pose a threat to the ecosystem if discharged without proper treatment. The existing biological treatments rarely meet the latest discharge limitations of 80 mg∙L^−1^ stated in the GB4287-2012 standard for dyeing and finishing wastewater. As a result, advanced oxidation techniques are frequently employed for further treatment of bio-treated dyeing and finishing wastewater (BDFW) in various researches. Soares *et al*.^1^ reported that photocatalytic Fenton is an efficient method for decolourisation of BDFW and Wu *et al*.^2^ showed that catalytic ozonation can effectively reduce the COD as well as the dissolved organic carbon (DOC) of BDFW. Holkar *et al*.^3^ reviewed combined biological and chemical process including ozonation, photo-Fenton, electrochemical, photocatalytic, H_2_O_2_ and zero valent iron with ultrasonic irradiation for textile wastewater treatment.

Fenton’s reagent, which consists of ferrous ion (Fe^2+^) and hydrogen peroxide (H_2_O_2_), has been widely used to decompose a broad variety of non-biodegradable compounds, including aromatic amines^4^, phenols^5^, dyes^6^ and antibiotics^7^. The removal and conversion of pollutants can be achieved through Fenton oxidation and coagulation. The mechanism of Fenton oxidation is commonly believed to be based on the reaction scheme proposed by Haber and Weiss^8^ and further modified by Walling^9^. The key oxidant ·OH, which attacks the organic compounds and decomposes them chemically, is triggered by the reaction between H_2_O_2_ and Fe^2+^ at acidic pH, whereas ferric ions are formed during redox reactions and help to remove the remaining pollutants through coagulation and precipitation at neutral pH. The traditional Fenton’s process usually has a longer hydraulic retention time (HRT) of 0.5–2.0 h^10^, and needs several tanks for pH adjustment, chemical addition, oxidation reaction, sedimentation etc., which requires additional space and higher capital cost. A new integrated rapid deep treatment process is preferred for industrial application.

In recent years, increasing attention has been paid to a shorter Fenton’s reaction time. Melgoza *et al*.^11^ reported a removal efficiency of close to 98.6% for methylene blue in the first 10 min. Giri and Golder^7^ also reported 94.2% decomposition of ciprofloxacin in the first 5 min. Wang even observed more than 40% degradation of 2, 4-dinitrophenol within the first second^12^. Many kinetic studies have been conducted using conventional methods to elucidate the Fenton oxidation mechanism^13–15^. However, most of the studies were sampled at minute-scale intervals^16^, and the fitted kinetic curve can deviate significantly from the actual experimental curve. Hence, special equipment with second-scale sampling intervals is necessary for a more detailed kinetic study of Fenton oxidation.

Stopped-flow spectrophotometer (SFS) has been frequently used to study rapid chemical processes, such as chlorination^17^, adsorption^18^ and decolorization of azo dyes by crude manganese peroxidase^19–21^. SFS has proved to be highly efficient in investigating the degradation of organic pollutants in less than 20 s reaction time^20^.

SFS was also applied to study the degradation mechanism and reaction kinetics of 4-chlorophenol in Fenton oxidation process^22^. Moreover, the fact that very small volumes (100–120 μL) of solutions were injected each time increased the difficulty of its study since the reactions could not be terminated immediately for COD analysis. The aim of this study is first to obtain a detailed kinetic model of Fenton oxidation and the corresponding kinetic constants. The rapid reaction between Fe^2+^ and H_2_O_2_ was analysed using SFS at second-scale intervals. In addition, a full-scale plant was designed and operated based on results of stopped-flow study for tertiary treatment of the effluent of BDFW. In the full-scale plant, Fenton oxidation was conducted in a pipeline reactor coupled with shallow air floatation. Compared to conventional Fenton reactors, which require the construction of dosing tanks for the adjustment of the pH and occupy a large land^10^, the upgraded technology has the advantage of reaching the same removal efficiency at much lower investment and operating cost in significantly shorter reaction time. Furthermore, this is the first study that proposed the application of rapid Fenton oxidation as a tertiary process for BDFW in a full-scale plant.

## Materials and Methods

### Materials and reagents

**Figure 1:**
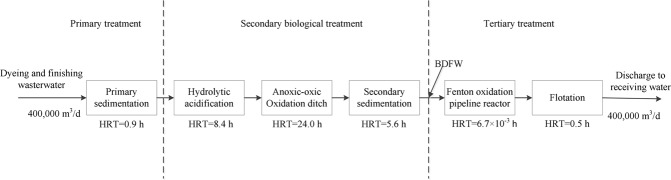
Flow chart of the full-scale treatment plant.

The effluent of BDFW was obtained from a full-scale wastewater treatment plant located in a typical dyeing and finishing industry cluster in southeast China. With a treatment capacity of 400,000 m^3^·d^−1^, the plant handles centralized treatment of wastewater from more than one hundred mills in this industry cluster. As shown in Fig. 1, the mixed dyeing and finishing wastewater flows through a typical secondary biological treatment and advanced oxidation process, namely primary sedimentation, hydrolytic acidification, anoxic/oxic oxidation ditch, secondary sedimentation, as well as Fenton oxidation and floatation in sequence, with corresponding HRTs of 0.9 h, 8.4 h, 24.0 h, 5.6 h, 6.7 × 10^−3^ h and 0.5 h. The Fenton oxidation process has been applied since 2014. Temperature ranged 20–35 °C without control.

All the chemicals used were analytical grade or HPLC grade and were used without any further purification. The luminescent bacteria, *Photobacterium phosphoreum*, were purchased from Hamamatsu Photon Techniques Inc., China. Ferrous sulphate (FeSO_4_·7H_2_O), hydrogen peroxide (H_2_O_2_ 30%) and other chemicals were purchased from Sinopharm Chemical Reagents Co., Ltd, China. Rhodamine B was purchased from Aladdin Industrial Corporation. Ultrapure water was used for solution preparation.

### Experimental procedure

#### Rapid Fenton reaction of synthetic wastewater containing methylene blue and rhodamine B using SFS

Rapid Fenton reaction was studied in aqueous phase using SFS (SFS, Model SX20, Applied Photophysics Ltd., UK). Two dyes, methylene blue and rhodamine B, were selected as the test pollutants in the SFS experiments because they can be detected *in situ* by UV-visible spectrophotometry (Ultraviolet and visible spectrophotometry). The initial content of the test dyes was 10 mg·L^−1^. A fixed H_2_O_2_ dosage (40 mg·L^−1^) and four different H_2_O_2_/Fe^2+^ molar ratios (4:1, 2:1, 1:1 and 1:2) were tested. The degradation kinetics of the dyes in Fenton reaction at initial pH, pH_ini_ of 2.0 were assessed at 293 ± 2 K. The pH_ini_ was specially adjusted to 2.0 to avoid spontaneous oxidation of Fe^2+^ with O_2_ and coagulation process inside the cuvette, which would block the tiny pipe of the SFS. Before the stopped-flow kinetic experiments, two working solutions (dye + H_2_SO_4_ + FeSO_4_ and H_2_O_2_ + H_2_SO_4_) were prepared. Reactions were initiated by simultaneously injecting an equal volume of the two working solutions into the optical cell of the SFS using two automatic syringes driven by compressed nitrogen. An UV−visible spectrophotometer was used as detectors with a 150 W xenon lamp as the light source. An HP computer workstation was used to control the SFS and acquire kinetic data. A blank experiment^21^ was conducted as ferric or ferrous ion has significant adsorption, which can interfere with monitoring of the dye concentration.

#### Batch experiment of Fenton oxidation for real BDFW

SFS is suitable for spectrophotometric study of rapid processes at second scale intervals, however, it cannot be coupled to separation devices to analyse multiple organic pollutants^23^. Batch experiment was conducted using a 500 mL beaker as the reactor. Real BDFW wastewater of 300 mL was added into the reactor. The initial pH was adjusted using H_2_SO_4_ (6 M) and NaOH (2 M). Then, the required amounts of FeSO_4_·7H_2_O and H_2_O_2_ (30%) were added to the reactor, which was continuously mixed for 1–60 min at room temperature (293 ± 2 K) using a magnetic stirrer. The reactor was covered with aluminium foil during the reaction to prevent light decomposition of H_2_O_2_. After the reaction, NaOH (2 M) was immediately added into the sample to adjust the pH to 8.0–8.5 for precipitation and the supernatants were sampled for SCOD (soluble chemical oxygen demand) analysis. NaOH was selected as the Fenton reaction terminator because Na_2_S_2_O_3_ as a reducing agent affects SCOD analysis. In BDFW, the concentrations of proteins and polysaccharides were 55.2 mg·L^−1^ and 19.2 mg·L^−1^, respectively, accounting for 63% of the SCOD based on a stoichiometric conversion factor of 1.5 and 1.2^2,24^. As the soluble microbial products (SMPs) contributed significantly to the COD. The SCOD, dissolved organic carbon (DOC), protein and polysaccharides were analysed.

#### Full-scale test of rapid Fenton reaction with real BDFW wastewater

BDFW, Fenton effluent (continuous-flow reaction terminated by adjusting pH to 9.0 with NaOH) and air floatation effluent collected from the full-scale plant with a treatment capacity of 400,000 m^3^·d^−1^ run continuously from 2014 were sampled for the analysis of COD, five-day biochemical oxygen demand (BOD_5_), acute toxicity, colour, total phosphate (TP), NH_3_-N, and pH analyses, etc.. The samples were filtrated through a 0.22 mm membrane for SCOD, DOC, total nitrogen (TN), protein, polysaccharides,, molecular weight distribution (MWD), three-dimensional excitation emission matrix fluorescence spectroscopy (3DEEM), GC-MS, and UHPLC-QTOF analyses. The samples for the 3DEEM analysis were diluted five times.

All experiments were conducted at least in triplicate, and the data were averaged with standard deviations <5%.

### Analytical methods

In the SFS experiments, the concentrations of methylene blue and rhodamine B were continuously monitored at 664 nm and 554 nm, respectively using a UV-visible spectrophotometer. The interference of Fe^2+^/H_2_O_2_ at UV absorbance of 664 nm was manually eliminated for methylene blue detection. The pH value was measured using a pH meter (Sartorius PB-10, Germany) calibrated with proper buffer solution (pH 4.00, 6.86, and 9.18) to ensure its accuracy. The DOC and TN were measured using a TOC analyser (Shimadzu TOC-L, Japan). The BOD_5_ was measured using a manometric respirometric BOD OxiTop^®^ analyser. The analyses of COD, SCOD, colour, TP and NH_3_-N were performed in accordance with standard methods^25^. The protein concentration was determined using the Lowry method^26^, and the polysaccharide concentration was determined using the anthrone colorimetric assay^27^. The MW distribution was analysed using gel chromatography (Agilent 1200, USA with Agilent PL aquagel-OH 30 column) according to the procedure reported by Yan *et al*.^28^. The acute toxicity was assessed using bright luminescent bacteria acute toxicity analysis according to the method reported by Liu *et al*.^29^ and international standard ISO 11348-3^30^. The 3DEEM was determined using a fluorescence spectrometer (HORIBA Jobin Yvon FluoroMax-4, France). The organic species were analysed using GC-MS (Shimadzu GCMS-QP2010 SE, Japan with HP5-MS column). The sample preparation and analysis method for GC-MS were performed according to the USEPA method 625 (USEPA, 2005). The peak area and relative peak area (%) for each compound were calculated according to the protocol reported by Zhuang *et al*.^31^. The variations of organic pollutant species were identified using UHPLC-QTOF (Agilent 1290 UHPLC, Agilent 6540 QTOF, USA with Agilent ZORBAX SB-C18 HD column). The UHPLC-QTOF-MS data were first analysed using Agilent Mass Profinder software to remove background noise and unrelated ions. All the features with abundance of >1000 were extracted in this process^32^.

## Results and Discussion

### Kinetic modelling of decolorization using SFS

**Figure 2:**
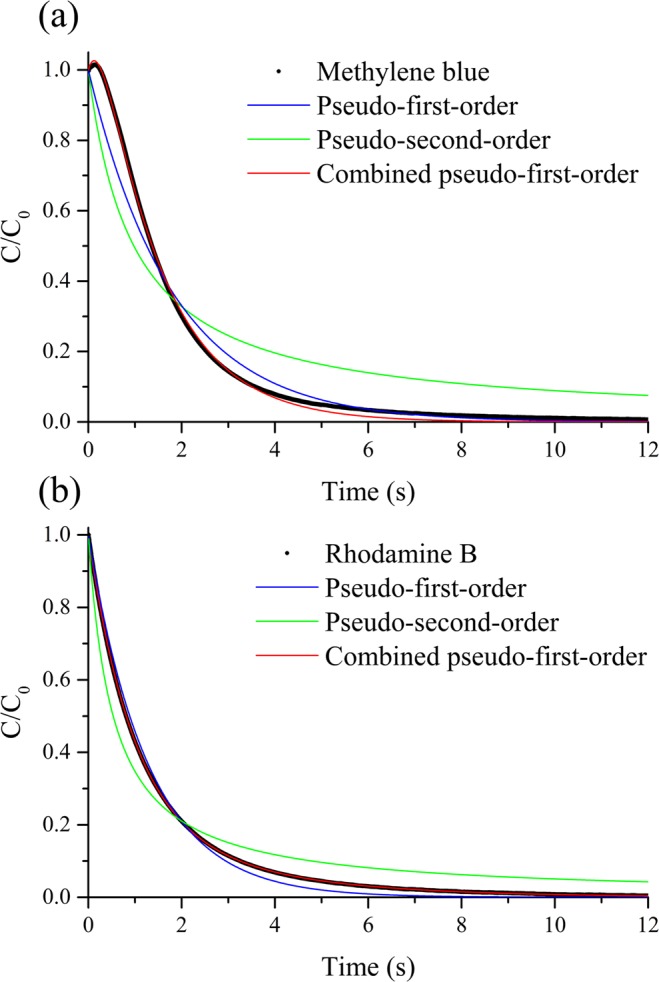
Variations of concentration with time for (**a**) methylene blue and (**b**) rhodamine B, as well as corresponding kinetic modelling for Fenton oxidation in SFS. Experimental conditions: [methylene blue]_0_ or [rhodamine B]_0_ = 10 mg·L^−1^, H_2_O_2_ dosage = 40 mg·L^−1^, [H_2_O_2_]:[Fe^2+^] mole ratio = 1:2, pH_0 = _2.0, T = 293 ± 2 K, measuring frequency = 5000/second.

Figure 2 shows the dotted concentration variations curves of methylene blue and rhodamine B with time in Fenton oxidation using SFS. The dosage of H_2_O_2_ was selected considering that a good level of hydrogen peroxide for mineralization was expected to be calculated 1–2 times the stoichiometric requirements (1 g COD = 0.03125 mol O_2_ = 0.0625 mol H_2_O_2_)^33,34^. The fastest degradation was achieved at a molar ratio of 1:2 (H_2_O_2_/Fe^2+^). Both dyes were below the detection limits after approximately 10 s of reaction, and 60–80% of decolorization occurred within the first second. Wang^12^ reported that the first second of reaction contributes to more than 40% of the total degradation, which agrees with the results from this study. The entire removal process can be categorised into two regimes^35^, namely a rapid decay of test pollutants in the first 2 s, followed by a much slower retardation stage. The first fast reactive stage, referred to as the Fe^2+^/H_2_O_2_ stage, was initiated by the catalytic formation of ·OH^36^

1 $$F{e}^{2+}+{H}_{2}{O}_{2}\to HO\cdot +F{e}^{3+}+O{H}^{-}\,{{\rm{k}}}_{1}=76{M}^{-1}{s}^{-1}$$

The Fe^3+^ produced in the first stage may also react with H_2_O_2_ to form weaker radicals such as ·OOH and cause a second decomposition process for the test pollutant, as given in Eq. (2):

2 $$F{e}^{3+}+{H}_{2}{O}_{2}\to HOO\cdot +F{e}^{2+}+{H}^{+}\,{{\rm{k}}}_{2}=0.001-0.01{M}^{-1}{s}^{-1}$$

The second retardation stage, referred to as the Fe^3+^/H_2_O_2_ stage, was likely due to the depletion of ·OH in the solution and the slow rate of ·OOH formation^14^.

Despite the higher number of steps involved in the Fenton reaction, the oxidation process can be simplified by the following pseudo *n*th order reaction kinetics

3 $$-\frac{dC}{dt}=k{C}^{n}$$

where *C* represents the concentration of the organic pollutant, *n* is the order of the reaction, *k* is the apparent reaction rate constant and *t* is the time. For a pseudo first order reaction, the integrated form of Eq. (3) is

4 $$\frac{C}{{C}_{0}}={e}^{-kt}$$

in which *C*_0_ is the initial concentration of the organic pollutant. For a pseudo second order reaction, the integrated equation becomes

5 $$\frac{C}{{C}_{0}}=\frac{1}{k{C}_{0}t+1}$$

The results of the pseudo first order and pseudo second order models are plotted in Fig. 2 and the parameters obtained from the curve fitting are presented in Table 1. Both models exhibited poor correlations (R^2^ < 0.97) except for pseudo first order for rhodamine B (R^2^ = 0.992), which contradicts the conclusion from previous studies that the degradation of dyes by Fenton oxidation followed the pseudo first order kinetics^10,37,38^. A possible reason for this contradiction is that these studies considered only the initial stage of degradation rather than the entire process.

**Table 1:** Kinetics parameters for Fenton oxidation of dyes in SFS.

Dye	Pseudo first order		Pseudo second order		Combined pseudo first order			Two-stage		
	*k* (s^−1^)	R^2^	*k* (L·mg^−1^·s^−1^)	R^2^	*k*_1_ (s^−1^)	*k*_2_ (s^−1^)	R^2^	m(/)	b(s^−1^)	R^2^
Methylene blue	0.5537	0.9632	0.1025	0.8450	0.7573	3.6850	0.9991	1.828	1.994	0.9261
Rhodamine B	0.7809	0.9918	0.1877	0.9246	0.3406	1.0563	0.9999	1.0237	1.0217	0.8860

Experimental conditions in SFS: [methylene blue]_0_ or [rhodamine B]_0_ = 10 mg·L^−1^, H_2_O_2_ dosage = 40 mg·L^−1^, [H_2_O_2_]: [Fe^2+^] mole ratio = 1:2, pH_0_ = 2.0, T = 293 ± 2 K.

The combined pseudo first order model fits the decrease of both methylene blue and rhodamine B with very high regression coefficients (R^2^ = 0.9991 and 0.9999). The combined pseudo first order model was proposed by Wang^14^ based on the two-stage mechanism that two parallel reactions shown in Eqs (1) and (2) are responsible for dye degradation.

6 $${C}_{0}={C}_{10}{e}^{-{k}_{1}t}+{C}_{20}{e}^{-{k}_{2}t}$$

7 $${C}_{0}={C}_{10}+{C}_{20}$$

where *C*_10_ and *C*_20_ are the initial dye concentrations of the two independent pseudo first order reactions, and *k*_1_ and *k*_2_ are the apparent reaction rate constants, respectively. Using Eq. (7), Eq. (6) can be transformed into Eq. (8) as follows

8 $$\frac{{\rm{C}}}{{{\rm{C}}}_{0}}=\frac{{{\rm{C}}}_{10}}{{{\rm{C}}}_{0}}{{\rm{e}}}^{-{{\rm{k}}}_{1}{\rm{t}}}+(1-\frac{{{\rm{C}}}_{10}}{{{\rm{C}}}_{0}}){{\rm{e}}}^{-{{\rm{k}}}_{2}{\rm{t}}}$$

Chu^39^ and Behnajady^40^ proposed an approximate two-stage kinetics model, as given in Eq. (9).

9 $$\frac{C}{{C}_{0}}=1-\frac{t}{m+bt}$$

The R^2^ values of this kinetics model for methylene blue and rhodamine B were 0.881 and 0.916, which were lower than those of the combined pseudo first order model.

### Kinetic modelling of real BDFW by fenton oxidation in batch test

As a result of the heterogeneous composition of BDFW and the complexity of intermediates formed during the Fenton oxidation, it is virtually impossible to carry out a detailed kinetic study with each individual reaction. An approximate kinetic study based on SCOD, DOC and SMPs was carried out to represent the overall organic matters.

**Figure 3:**
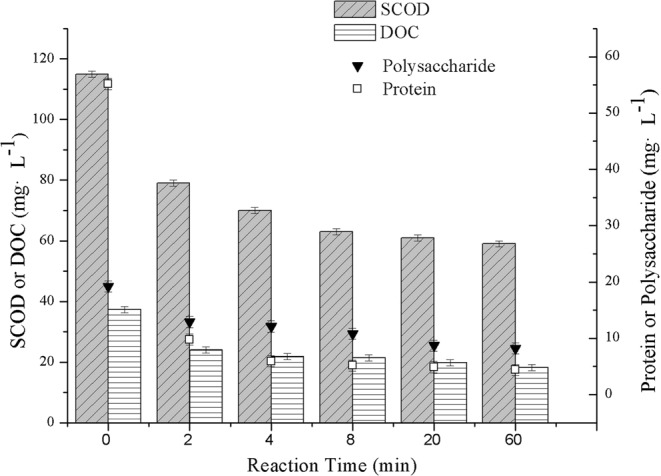
Variations of SCOD, DOC and SMPs in real BDFW with time by Fenton oxidation in batch test. Experimental conditions: H_2_O_2_ dosage = 200 mg·L^−1^, [H_2_O_2_]:[Fe^2+^] mole ratio = 1:2, pH_0_ = 4.0, T = 293 ± 2 K.

Figure 3 shows variations of SCOD and DOC levels as well as protein and polysaccharides concentration in real BDFW during Fenton oxidation. The SCOD in BDFW decreased significantly during the first 2 min, accounting for 64% of the total removal, and as the reaction time increased, the curve approached a plateau. Altogether, 82% of the proteins and 33% of the polysaccharides were removed in the first 2 min. The degradation rate of SMPs was slower under the interference of complex components. The removal efficiency of polysaccharides was only 63% after 60 min of reaction, which was probably because the polysaccharides in BDFW were more difficult to decompose than starch.

The reduction in SCOD and DOC both followed the combined pseudo first order kinetics well (R^2^ > 0.999):

10 $$\frac{{\rm{SCOD}}}{{{\rm{SCOD}}}_{0}}=\frac{{{\rm{SCOD}}}_{10}}{{{\rm{SCOD}}}_{0}}{{\rm{e}}}^{-{{\rm{k}}}_{1}{\rm{t}}}+(1-\frac{{{\rm{SCOD}}}_{10}}{{{\rm{SCOD}}}_{0}}){{\rm{e}}}^{-{{\rm{k}}}_{2}{\rm{t}}}$$

11 $$\frac{{\rm{DOC}}}{{{\rm{DOC}}}_{0}}=\frac{{{\rm{DOC}}}_{10}}{{{\rm{DOC}}}_{0}}{{\rm{e}}}^{-{{\rm{k}}}_{1}{\rm{t}}}+(1-\frac{{{\rm{DOC}}}_{10}}{{{\rm{DOC}}}_{0}}){{\rm{e}}}^{-{{\rm{k}}}_{2}{\rm{t}}}$$

**Figure 4:**
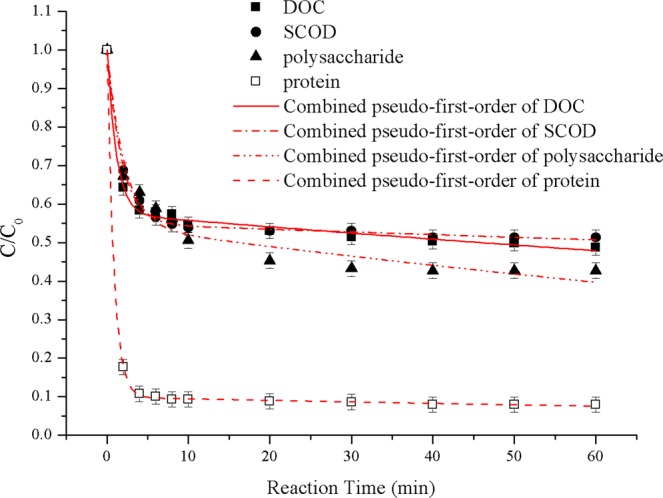
Kinetic modeling of SCOD, DOC and SMPs removal from real BDFW in batch test. Experimental conditions: H_2_O_2_ dosage = 200 mg·L^−1^, [H_2_O_2_]:[Fe^2+^] mole ratio = 1:2, pH_0_ = 4.0, T = 293 ± 2 K.

As shown in Fig. 4, the combined pseudo first order model for SCOD and DOC fit the curves well (R^2^ > 0.997), and the corresponding kinetic equations of SCOD and DOC are given in Eqs (12) and (13), respectively.

12 $$\frac{{\rm{SCOD}}}{{{\rm{SCOD}}}_{0}}=0.451{{\rm{e}}}^{-0.5531{\rm{t}}}+0.549{{\rm{e}}}^{-0.00132{\rm{t}}}$$

13 $$\frac{{\rm{DOC}}}{{{\rm{DOC}}}_{0}}=0.4301{{\rm{e}}}^{-0.8408{\rm{t}}}+0.5699{{\rm{e}}}^{-0.00284{\rm{t}}}$$

In a previous Fenton kinetics study, Lucas and Peres^33^ reported that COD decreased occurred principally at the initial period (10–20 min). However, COD levels in less than 10 min were left unmeasured. The decrease rule of SCOD and DOC in real BDFW during Fenton oxidation process in batch test fit well for both the combined pseudo-first-order kinetics model (R^2^ = 0.997 and 0.997) proposed by Wang^14^ and the two-stage kinetics model (R^2^ = 0.996 and 0.991) proposed by Chu^39^ and Behnajady^40^. The results in Figs 3 and 4 indicate that the Fenton treatment was also efficient for the removal of organic matters in less than 2 min.

### Full-scale application of rapid Fenton oxidation for BDFW at second scale intervals

**Figure 5:**
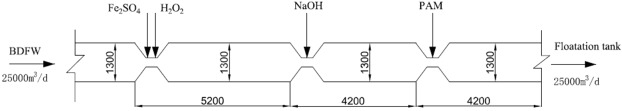
Schematic diagram of the rapid Fenton process using pipeline reactor at second-scale intervals.

Rapid Fenton treatment of the BDFW in a full-scale plant with capacity of 400,000 m^3^·d^−1^ have been performed continuously in 16 pipeline reactor systems since 2014. As shown in Fig. 5, the total pipeline length, volume and treatment capacity of each system were 13.6 m, 18.32 m^3^ and 25,000 m^3^·d^−1^, respectively. As the effluent from the secondary settler flowed into the pipe, acidified ferrous sulphate solution and hydrogen peroxide solution were added into the pipeline reactor in sequence through a pipe dosing dispenser. After approximately 24 s of Fenton oxidation in the 6.9 m^3^ pipeline reactor, the reaction was then terminated by adjusting the pH to above 6 using liquid alkali. Then, the flocs were formed by dosing polyacrylamide (PAM) solution. Afterwards, the mixed liquor flowed into a shallow air floatation tank with an effective water depth of 0.8 m.

#### Conventional parameters

Conventional parameters were measured in the effluents from different stages of the rapid Fenton process of BDFW. The BDFW was sampled from the secondary settler; the Fenton effluent was sampled from the mixed liquor after dosing PAM solution, whereas the floatation effluent was sampled from the floatation tank. The most appropriate reaction condition was determined by batch experiments owing to treatment cost concern, as described in the Supplementary Information. As presented in Table 2, the COD level decreased from 140 mg·L^−1^ to 77 mg·L^−1^ in the Fenton effluent with a removal efficiency of 45%, which meets the discharge limitation of 80 mg·L^−1^. The SCOD level decreased from 110 mg·L^−1^ to 71 mg·L^−1^ with a removal efficiency of 35%, and the DOC level decreased from 35 mg·L^−1^ to 26 mg·L^−1^ with a removal efficiency of 26%. When the kinetic model in Eq. (12) was used, the time taken to reach the same SCOD removal efficiency was 2.75 min. This is mainly due to lack of SCOD data at Fenton reaction time less than 2 min. Thus, the kinetic model could not accurately predict the second-scale SCOD removal efficiency in BDFW. Another possible reason is the different agitation intensities between batch and full-scale applications. The removal of soluble organic matter contributed mostly to the COD reduction. The inhibitory effect decreased from 51% to 31%, which indicates that part of the high toxic pollutants was degraded into less toxic or non-toxic substances. The BOD_5_, TN, NH_3_-N and TP values also decreased. All the conventional parameters met the national discharge standard, namely GB 4287–2012, except TN. Further biological treatment may be necessary for the standard discharge of TN.

**Table 2:** Conventional parameters of the effluents from different stages of the rapid Fenton process of BDFW at second-scale intervals.

Parameter		Average value ± standard deviation		
		Secondary effluent	Fenton effluent	Floatation effluent
COD	(mg·L^−1^)	140 ± 3	77 ± 3	76 ± 3
	removal efficiency	/	45% ± 2%	46% ± 2%
SCOD	(mg·L^−1^)	110 ± 3	71 ± 3	70 ± 3
	removal efficiency	/	35% ± 3%	36% ± 3%
DOC	(mg-C·L^−1^)	35 ± 2	26 ± 2	25 ± 2
	removal efficiency	/	26% ± 3%	29% ± 3%
BOD_5_	(mg·L^−1^)	3 ± 1	1 ± 1	2 ± 1
	removal efficiency	/	67% ± 33%	33% ± 33%
TN	(mg-N·L^−1^)	48 ± 2	47 ± 2	46 ± 2
	removal efficiency	/	2% ± 2%	4% ± 2%
NH_3_-N	(mg-N·L^−1^)	0.04 ± 0.01	0.03 ± 0.01	0.02 ± 0.01
	removal efficiency	/	25% ± 25%	50% ± 25%
TP	(mg-P·L^−1^)	4.19 ± 0.05	1.14 ± 0.05	1.21 ± 0.05
	removal efficiency	/	73% ± 1%	71% ± 1%
Inhibitory effect (%)		51	31	30

Experimental conditions: H_2_O_2_ dosage = 128 mg·L^−1^, [H_2_O_2_]:[Fe^2+^] mole ratio = 1:2, reaction time = 24 s, pH = 3–4.

#### 3DEEM fluorescence

**Figure 6:**
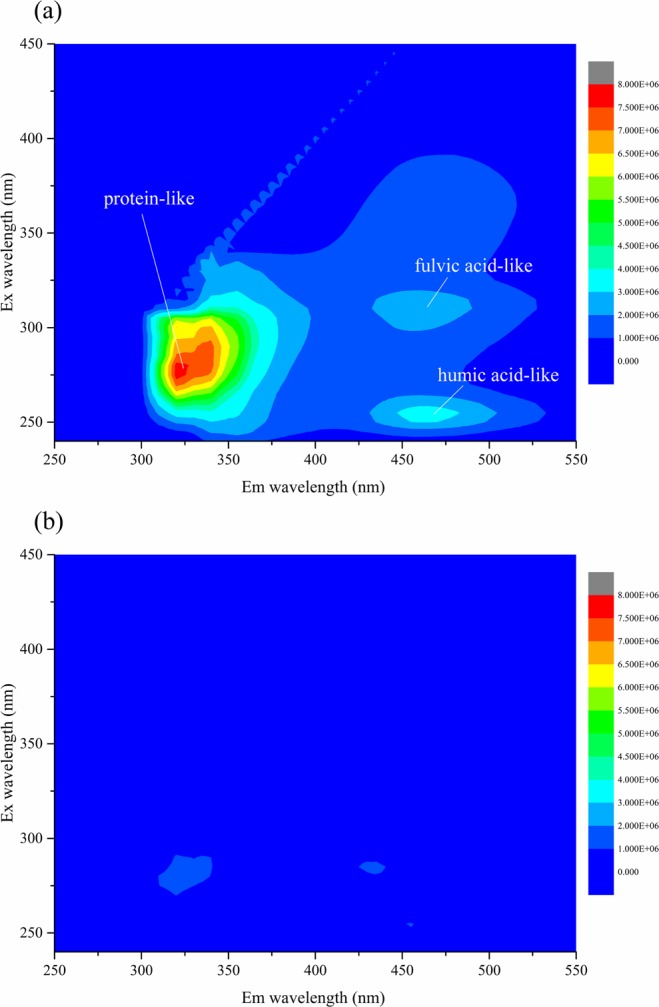
Variations in 3DEEM before and after the rapid Fenton process in full-scale plant: (**a**) BDFW, (**b**) Fenton. Experimental conditions: H_2_O_2_ dosage = 128 mg·L^−1^, [H_2_O_2_]:[Fe^2+^] mole ratio = 1:2, reaction time = 24 s, pH = 3–4.

Figure 6 shows the three fluorescence peaks of the BDFW sample, which are similar to results obtained by Wu *et al*.^2^ (i.e., Flu. 1 for protein-like (Ex/Em = 270/315 nm), classified as SMP-like, Flu. 2 for humic acid-like (Ex/Em = 290/450 nm) and Flu. 3 for fulvic acid-like (Ex/Em = 250/460 nm))^41^. It can be observed that most of the effluent organic matter were SMP-like substances, which is typical for BDFW^2,42^. After the Fenton process, none of the three peaks were detected in the effluent, indicating that rapid Fenton treatment can remove the natural fluorescent substances in BDFW completely.

#### MW distribution

Two molecule fractions were contained in the BDFW, similar to results obtained by Wu *et al*.^2^ (i.e., Peak 1 for MW 2.1–29 kDa (86%) and Peak 2 for MW 0.05–2.1 kDa (14%)). After the rapid Fenton treatment, the relative peak area of Peak 1 increased from 86% to 94% with a decrease in that of Peak 2, suggesting the mineralization of low molecular weight acids. Specific data can be found in Supplementary Information.

#### Variations in organic pollutant species

To detect variations in organic pollutant species, UHPLC-QTOF technology and GC-MS technology were used. The UHPLC-QTOF method was performed in both positive ionization (PI) and negative ionization (NI) modes. A recursive feature extraction algorithm (Agilent Profinder 8.0) was applied, and 488 features in the positive mode and 507 features in the negative mode were extracted from the BDFW, as presented in Table 3. After the rapid Fenton treatment, the number of organic species decreased by 36 in the negative mode and 15 in the positive mode. In both modes, the relative peak area (RPA) of strong polar species (retention time ≤2 min) increased, whereas that of medium or weak polar species (retention time >2 min) decreased. These results suggest that medium or weak polar organic pollutants were removed effectively, but the part of them removed were converted into polar organic pollutants in rapid Fenton oxidation. The removal of organic pollutants was occurring but was limited even if the discharged standards were reached.

**Table 3:** Variations in the number of organic species detected by UHPLC-QTOF.

	Negative			Positive		
	Total	RT ≤ 2 min	RT > 2 min	Total	RT ≤ 2 min	RT > 2 min
BDFW	507	283	224	488	129	359
	RPA (%)	41.08	58.92	/	14.55	85.45
Fenton	471	269	202	473	127	346
	RPA (%)	51.38	48.62	/	21.08	78.92

Experimental conditions: H_2_O_2_ dosage = 128 mg·L^−1^, [H_2_O_2_]:[Fe^2+^] mole ratio = 1:2, reaction time = 24 s.

Table 4 lists the main organic pollutant species detected with MW < 500 Da and relative peak areas >0.1% using GC-MS. Di-methyldodecylamine, the main pollutant in BDFW, is a type of textile auxiliaries and dye intermediates. Other pollutants include industrial chemicals (mainly alkanes), plasticizers (dibutyl phthalate, diisobutyl phthalate) and others (decane-1,10-diol). After the rapid Fenton treatment, 34% of the Di-methyldodecylamine amount was reduced. Various other pollutants, such as 2-bromo dodecane and decane, 3,8-dimethyl- were not detected in the effluent. Bis(2-ethylhexyl) phthalate was detected in the Fenton effluent but not in the BDFW, and it has a similar molecular structure to diisobutyl phthalate, which could probably be its origin. The peak area of di-methyldodecylamine, dibutyl phthalate and tetracosane decreased slightly. In Fenton oxidation effluent, 40.59% of total peak area of GC-MS was reduced, and the peak area of a variety of long-chain organic pollutants decreased and was even undetected, replaced by short-chain organic pollutants. Fenton process removed some organic pollutants and convert long-chain organic compounds into short-chain organic compounds. The removal of organic pollutants was occurring but was limited.

**Table 4:** Main organic pollutant species detected by GC-MS before and after the rapid Fenton process.

Species	BDFW		Fenton effluent	
	PA	RPA(%)	PA	RPA(%)
**Textile auxiliaries and dye intermediates**				
Di-methyldodecylamine	2099258	20.68	1380279	22.89
Phenol, 3, 5 – bis (1,1-dimethylethyl) -	55496	0.55	303683	5.03
Hexadecanoic acid	324629	3.20	596568	9.89
**Industrial chemicals**				
Dodecane	141542	1.39	218957	3.63
2-Bromo dodecane	47943	0.47	ND	ND
Decane, 3,8-dimethyl-	59375	0.59	ND	ND
Octane, 3, 4, 5, 6 -tetramethyl-	65510	0.65	ND	ND
Octadecane	ND	ND	28176	0.47
Octane, 3-ethyl-2,7-dimethyl-	ND	ND	50850	0.84
Nonadecane	27941	0.28	ND	ND
Tetracosane	56120	0.55	53705	0.89
Pentane, 2,2,4,4-tetramethyl-3-methoxy-	67953	0.67	ND	ND
1-Pentadecanamine, N,N-dimethyl-	210223	2.07	ND	ND
N-methyl-N-benzyldodecanamine	ND	ND	193060	3.20
Octanamide, N, N -dimethyl-	29504	0.29	ND	ND
**Plasticizers**				
Dibutyl phthalate	295691	2.91	246103	4.08
Diisobutyl phthalate	140039	1.38	437649	7.25
Octadecanoic acid	ND	ND	282658	4.68
Bis(2-ethylhexyl) phthalate	ND	ND	174423	2.89
**Others**				
Decane-1,10-diol	42260	0.42	ND	ND
Nonane, 4,5-dimethyl-	29042	0.29	ND	ND
Decyl acetate	48806	0.48	ND	ND
Z-10-Tetradecen-1-ol acetate	ND	ND	58613	0.97
Pentane, 2,3,3-trimethyl-	ND	ND	14306	0.24

#### Applicability of rapid Fenton oxidation for BDFW at second scale intervals

The traditional Fenton oxidation reactor is a mature craft. However, rapid Fenton oxidation reactor with a pipeline reactor at second-scale intervals, has advantage over traditional Fenton type in several aspects. HRT for rapid Fenton oxidation using pipeline reactor is only 24 s, which is significantly shorter than that of the traditional Fenton process with at least 15 minutes, greatly reducing the volume of reaction tank, resulting in great reduction of construction cost and land. In fact, the full scale rapid Fenton process at second-scale intervals using pipeline reactor in this research was built at the bottom of original gas flotation, so no another area was needed for these pipeline reactors. Only 7.46×10^5^ USD was spent for the construction of 16 pipeline reactors, each having a total volume of 18.04 m^3^ and 62.43 seconds of HRT as well as a volume of 6.9 m^3^ and 24 seconds of HRT of Fenton’s reaction for the treatment effluent of BDFW of 400,000 m^3^·d^−1^ in 2014. In addition, the run of Fenton reaction was trouble because many chemicals such as FeSO_4_, H_2_O_2_, NaOH, PAM were needed to be added into different stages. In traditional Fenton’s reaction, these chemicals were added and mixed in different reactors in sequence. However, those chemicals were added in sequence in pipeline reactor utilizing syphon principle, making the addition of chemicals become easy. The run of the rapid Fenton oxidation using pipeline reactor become convenient, saving electric and manual maintenance cost. The rapid Fenton oxidation using pipeline reactor was similar to traditional Fenton process in the cost of chemicals and sludge handling.

## Conclusions

The following conclusions are drawn from the findings of this study.

1. The decrease of methylene blue and rhodamine B by Fenton oxidation in SFS followed a two-stage pattern. The combined pseudo first order model fits the decrease curve with high regression coefficient (R^2^ > 0.999), whereas the pseudo first order and pseudo second order models exhibited poor correlation (R^2^ < 0.98).
2. For Fenton oxidation of real BDFW, the combined pseudo first order model also fits the COD/COD_0_ curve well (R^2^ > 0.999), and the corresponding kinetic constants were 0.0015 s^−1^ for *k*_1_ and 0.5423 s^−1^ for *k*_2_. The removal of COD in the initial 2 min accounted for 64% of the total removal.
3. The SMPs, which contributed greatly to the COD in real BDFW, had good removal efficiency in the rapid Fenton reaction. In total, 82% of the proteins and 33% of the polysaccharides were removed in the first 2 min.
4. The rapid Fenton treatment proved to be an effective technique for BDFW in a full-scale plant. Under appropriate operating conditions, the COD level decreased from 140 mg·L^−1^ to 77 mg·L^−1^, the SCOD level decreased from 110 mg·L^−1^ to 71 mg·L^−1^, and the DOC level decreased from 35 mg·L^−1^ to 26 mg·L^−1^.
5. The process of rapid Fenton reaction in pipeline reactors at second intervals, together with the process of adjusting the pH to neutral and dosing PAM solution in the pipeline reactor system significantly improved the efficiency by minimizing the volume of reactors and simplifying the operating procedures.
6. The inhibitory effect decreased from 51% to 31% after the rapid Fenton treatment, suggesting reduced toxicity. The 3DEEM demonstrated complete removal of natural fluorescent substances in BDFW. MW distribution showed the mineralization of low molecular weight acids.
7. The Fenton process exhibited efficient performance for the removal of both polar and non-polar organic pollutants. After the rapid Fenton treatment, the number of organic species decreased by 36 and 15 in the negative mode and positive mode, respectively. In both modes, the RPA of strong polar species (retention time ≤2 min) increased, whereas that of medium or weak polar species (retention time >2 min) decreased.
8. The main pollutants detected by GC-MS can be classified into four groups, namely textile auxiliaries and dye intermediates, industrial chemicals, plasticizers and others. It was found that 34% of the most abundant pollutant, di-methyldodecylamine, was removed, and various other pollutants were not detected in the effluent.
9. The rapid Fenton oxidation using pipeline reactor at second-scale intervals has advantages over the traditional Fenton reactor in terms of low land requirement, easy operation, less construction and maintenance cost.

## Supplementary information

Supplementary Info 1

Supplementary Info 2

Supplementary Info 3

Dataset 1A

Dataset 1B
