# Supplementary material for: A Rapid Fenton treatment of bio-treated dyeing and finishing wastewater at second-scale intervals: kinetics by stopped-flow technique and application in a full-scale plant
Source: Sci Rep. 2019 Jul 4;9:9689. doi: 10.1038/s41598-019-45948-9 (PMC6609656; doi:10.1038/s41598-019-45948-9)
Supplement: Supplementary file 1 — Supplementary Info 1 [file 41598_2019_45948_MOESM1_ESM.pdf]

Supplementary Information

for

A Rapid Fenton treatment of bio-treated dyeing and finishing wastewater at second-scale intervals: kinetics by stopped-flow technique and application in a full-scale plant

Yunlu Chen [a], Yunqin Cheng [a], Xiaohong Guan [b], Yan Liu [a,], Jianxin Nie [a], Chenxi Li [a]*

[a] Department of Environmental Science and Engineering, Fudan University, Shanghai 200433, China

[b] College of Environmental Science and Engineering, Tongji University, Shanghai 200092, China

**Corresponding author. Tel.: +86-21-6564-3894; fax: +86-21-6564-3597;*

Email: liuyan@fudan.edu.cn

1. Materials and Methods

1.1 Wastewater and reagents

Real wastewater was obtained from the effluent of a full-scale wastewater treatment plant located in a typical dyeing and finishing industry cluster in southeast China. The plant employed a typical secondary treatment process including primary sedimentation, hydrolytic acidification, oxidation ditch and secondary sedimentation in sequence. With a designed capacity of 400,000 $m^3 \cdot d^{-1}$, the plant handles centralized treatment of wastewater from more than one hundred dyeing and finishing factories in this industry cluster.

The chemicals used were analytical grade or above. The luminescent bacteria, *Photobacterium phosphoreum*, were purchased from Hamamatsu Photon Techniques Inc., China. Ferrous sulphate, hydrogen peroxide (30%) and other chemicals were purchased from Sinopharm Chemical Reagents Co., Ltd, China. The solutions were prepared using ultrapure water.

1.2 Experimental procedure

The Fenton's experiments were conducted in batch reactors composed of beaker and magnetic stirrer. The reactors were covered with aluminium foil to avoid possible influence of light. Both BDFW and H_2O_2 were adjusted to the target pH levels by adding H_2SO_4 or NaOH. Before the reaction, wastewater and ferrous sulphate were added into the reactor and mixed continuously using a magnetic stirrer. Then, the reaction was initiated by adding H_2O_2 into the reactor. Timekeeping and protection from light were initiated as soon as reagent addition has been completed. At the end of the pre-set reaction time, NaOH was immediately added into the reactor, which terminated the reaction by adjusting the pH to 8–8.5. Several parameters influence the pollutants removal efficiency of the Fenton's process, including the reaction time, H_2O_2 dosage, pH, the initial molar ratio of H_2O_2 and ferrous iron. To optimize the reaction condition, both orthogonal experiment and single factor experiment were conducted.

After 1 h precipitation, the supernatants were sampled for chemical oxygen demand (COD), five-day biochemical oxygen demand (BOD_5), acute toxicity, colour, total phosphate (TP), NH_3-N, total suspended solids (TSS) and pH analyses. Moreover, the samples were filtered using 0.22 mm membrane for soluble chemical oxygen demand (SCOD), dissolved organic carbon (DOC), total nitrogen (TN), soluble iron (SFe), remaining H_2O_2 and soluble microbial products (SMPs) analyses.

1.3 Analytical methods

The DOC and TN were determined using a TOC analyser (Shimadzu TOC-L, Japan). The BOD_5 was measured using the manometric respirometric BOD OxiTop® method. The protein concentration was determined using the Lowery method[1], and the polysaccharide concentration was determined using the anthrone colorimetric assay[2]. The COD, SCOD, colour, TP, NH_3-N, total iron (TFe), Fe^{2+} and TSS were determined using standard methods[3]. The pH value was measured using a pH meter (Sartorius PB-10, Germany).

2. Results and discussion

2.1 Characterization of BDFW

The BDFW was characterized and the related parameters are listed in Supplementary

Table S1. Several parameters such as COD, TN and TP did not meet the requirement of the discharge standard. The COD level was as high as 156 mg/L, which is almost double the discharge standard, whereas the BOD_5 level was only 1 mg/L. Thus, further bio-treatment is not suitable for COD reduction of secondary effluent, and advanced oxidation by Fenton reaction is needed.

Table S1 Water quality of BDFW and Fenton effluent for real BDFW in batch test

	BDFW	Fenton effluent (Supernatant)
pH	7.65±0.34	8~8.5
COD (mg/L)	156±3	68±3 (66±3)
SCOD (mg/L)	118±3	63±3 (61±3)
DOC (mg/L)	42±2	23±2
TP (mg/L)	3.05±0.07	0.34±0.06
TN (mg/L)	38±2	37±2
NH_3-N (mg/L)	1.78±0.05	2.61±0.05
Color	31±3	4±3
TSS (mg/L)	23±5	6±5
BOD_5(mg/L)	1±0.5	8±0.5
Protein(mg/L)	45.27	15.27
Polysaccharide(mg/L)	35.7	30.3

2.2 Effect of H_2O_2 dosage, initial pH, initial molar ratio of H_2O_2 and ferrous iron

2.2.1 Orthogonal experiment

The orthogonal experiment aims to determine the approximate optimum condition for the above parameters. The orthogonal array is presented in Supplementary Table S2 based on engineering application experience, and the detailed reaction conditions and results for each group are presented in Supplementary Table S3. The reaction time was 10 min to ensure fulfilment of the requirement of Fenton's reaction.

Table S2 Orthogonal array for real BDFW in batch test

	Initial pH	H_2O_2 (mg/L)	H_2O_2:Fe^{2+}
1	2	50	1:5
2	3	100	1:1
3	3.5	200	5:1
4	4	300	10:1

Table S3 Reaction conditions and results of orthogonal experiment for real BDFW in batch test

	Initial pH	H_2O_2 dosage (mg/L)	Ferrous sulfate dosage (mg/L)	Initial [H_2O_2]:[Fe^{2+}] mole ratio	SCOD (mg/L)	DOC (mg/L)
1	2	50	2043	1:5	74	29
2	2	100	817	1:1	86	33
3	2	200	327	5:1	88	39
4	2	300	245	10:1	90	39
5	3	50	409	1:1	74	27
6	3	100	4087	1:5	68	23
7	3	200	163	10:1	70	31
8	3	300	490	5:1	47	21
9	3.5	50	82	5:1	89	38
10	3.5	100	82	10:1	73	36
11	3.5	200	8175	1:5	71	23
12	3.5	300	2452	1:1	47	16
13	4	50	41	10:1	109	40
**14**	**4**	**100**	**163**	**5:1**	**66**	**26**
15	4	200	1635	1:1	47	16
16	4	300	12260	1:5	77	25

Only SCOD and DOC were analysed owing to the large amount of measurement task. As presented in Supplementary Table S3, seven groups of orthogonal experiments, including groups 6, 7, 8, 11, 12, 14, and 15 were effective for the reduction of the SCOD in the effluent, which can meet the discharge criteria.

For application of the Fenton's reaction (FR) for advanced treatment of BDFW, the process cost should be considered. This is determined by the price of hydrogen peroxide and ferrous sulphate, namely 850 yuan RMB/t and 150 yuan RMB/t in Lake Tai Basin area[4]. The disposal cost of dewatered iron sludge should also be taken into account, that is approximately1400 yuan RMB/t[5][6].

Groups 8, 12, and 15 had high removal percentage of more than 60% out of all the experiments. However, H_2O_2 was overdosed and the initial H_2O_2:Fe^{2+} mole ratio was lower than 1:1, which led to prior reaction of H_2O_2 and ferrous[7]. Thus, a certain amount of chemicals was wasted. For groups 6 and 11, the initial H_2O_2:Fe^{2+} mole ratio was 1:5, which produced a large amount of iron sludge. The SCOD removal efficiencies for groups 7 and 14 are similar, and the ferrous sludge content was acceptable. The 14th condition used half dosage of H_2O_2, which is more economical. Therefore, in the following the first single factor experiment, effect of H_2O_2 dosage, the reaction conditions were based on group 14 with initial pH of 4 and initial [H_2O_2]:[Fe^{2+}] mole ratio of 5:1.

2.2.2 Effect of H_2O_2 dosage

In the Fenton's reaction, H_2O_2 is the prime source of ·OH radicals, which contributes significantly to the removal of pollutants. To determine the relationship between SCOD and DOC removal and H_2O_2 dosage, a single factor experiment was conducted. Supplementary Figure S1 shows that with increasing H_2O_2 dosage, both SCOD and DOC exhibited a decreasing trend, and SCOD can meet the discharge standard when 120 mg/L H_2O_2 was added. In view of cost, 120 mg/L H_2O_2 was selected for following research about effect of [H_2O_2]:[Fe^{2+}] mole ratio and initial pH.

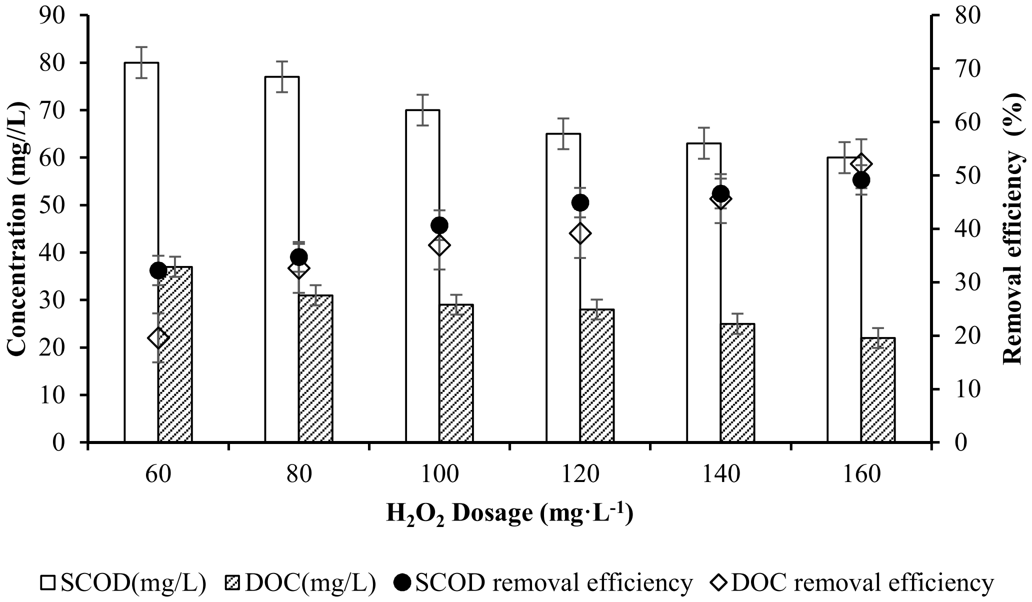

Figure S1 Effect of H_2O_2 dosage on SCOD and DOC removal for real BDFW by Fenton oxidation in batch test. Experimental conditions: $[H_2O_2]:[Fe^{2+}]$ mole ratio = 5:1, pH_0 = 4.0, T = 293 ± 2 K.

2.2.3 Effect of the initial molar ratio of H_2O_2 and ferrous iron

Ferrous ion is the catalyst for Fenton's reaction. As shown in Supplementary Figure S2, with increase in the molar ratio of H_2O_2 and ferrous ion from 1:2 to 8:1, the effluent SCOD and DOC decreased initially and then increased. The ratio of 1:1 had the highest SCOD removal efficiency of 41%. Moreover, the ratios of 1:1 and 1:2 had the highest DOC removal efficiency of 44% at H_2O_2 dosage of 120 mg/L and initial pH of 4 after 40-min reaction. At the same H_2O_2 dosage, a high amount of ferrous ion had a higher probability to react with ·OH radicals according to Supplementary equation (S1) such that the oxidation of organic pollutants was hindered. The following treatment of iron sludge, whose amount is associated with ferrous dosage, should also be considered. Initial molar ratio of 1:1 was selected for further research, where the SCOD was reduced to 52 mg/L.

$$Fe^{2+} + \cdot OH \rightarrow Fe^{3+} + HO^- \quad (S1)$$

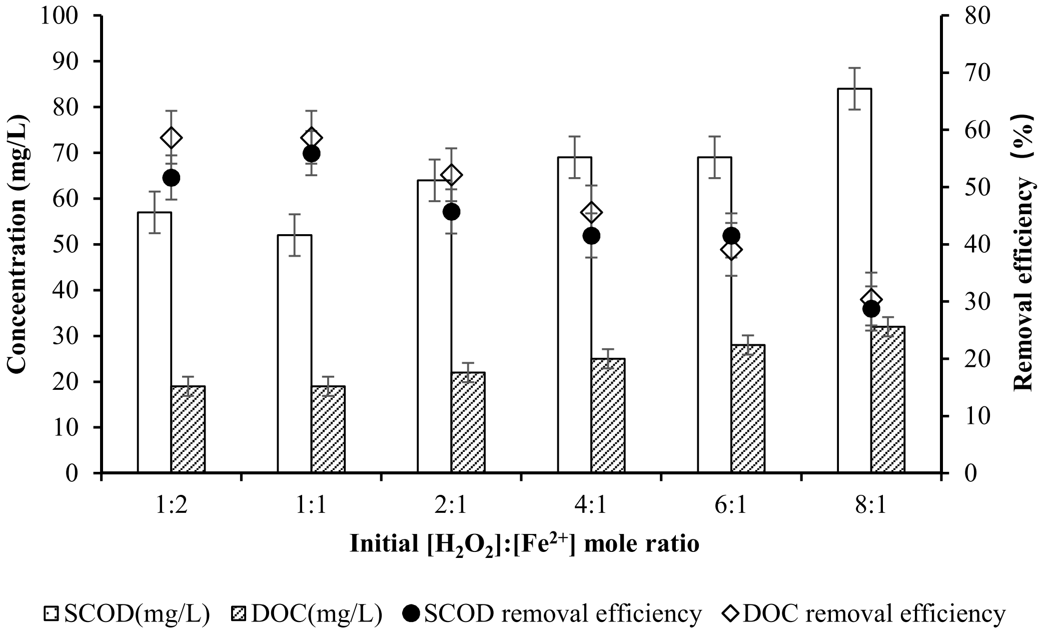

Figure S2 Effect of the initial [H_2O_2]:[Fe^{2+}] mole ratio on SCOD and DOC removal for real BDFW by Fenton oxidation in batch test. Experimental conditions: H_2O_2 dosage = 120 mg·L^{-1} , pH$_0$ = 4.0, T = 293 ± 2 K.

2.2.4 Effect of the initial pH

The initial pH was also observed to be a significant parameter in the Fenton's reaction[8]. Supplementary Figure S3 shows that the SCOD and DOC can be reduced to 63 mg/L and 23 mg/L, respectively under optimum condition with initial pH of 4. An increasing trend of SCOD and DOC removal was observed when the initial pH increased from 2.5 to 4, whereas the opposite trend was observed when the initial pH increased from 4 to 5.

The decreasing removal efficiency at lower pH is mainly due to the generation of [Fe(II)(H_2O)]$^{2+}$, which obstructs the reaction between ferrous ions and hydrogen peroxide to produce active ·OH radicals[9][10]. A low pH promotes the reactions in Supplementary equations (S1) and (S2), which consumes hydroxyl radicals and inhibits the reaction between Fe^{3+} and H_2O_2, contributing to the decrease in the SCOD and DOC removal efficiency[9].

$$H_2O_2 + HO\cdot \rightarrow HO_2\cdot + H_2O \qquad \text{(S2)}$$

High pH ensures the formation of ferric hydroxide and ferrous hydroxide, which leads to coagulating sedimentation. This results in reduced content of Fe ions in the reaction system[11] and obstructs the Fenton's reaction.

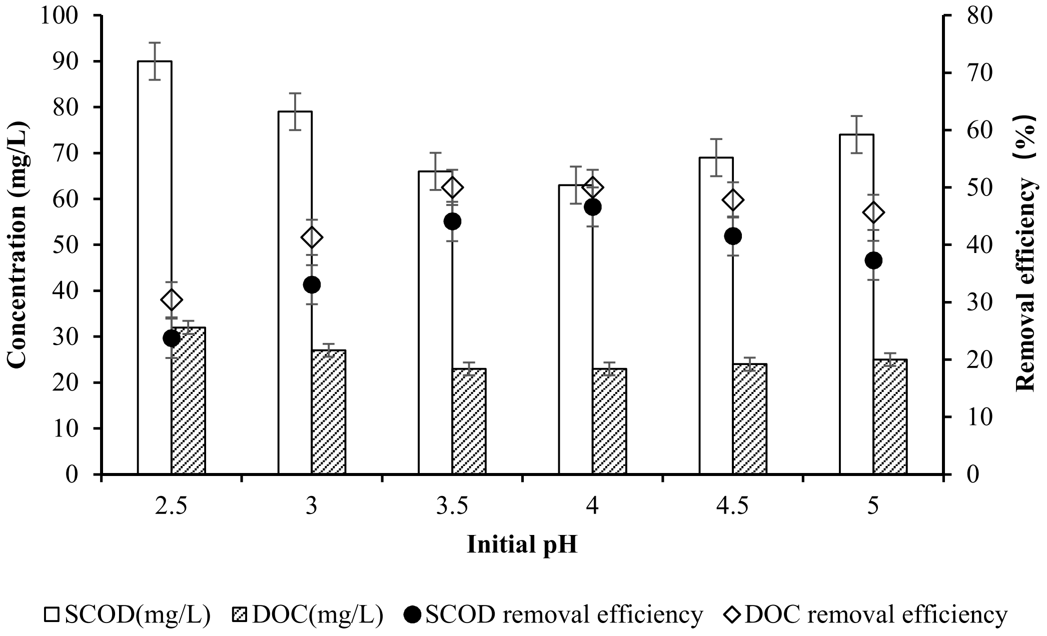

Figure S3 Effect of the initial pH on SCOD and DOC removal for real BDFW by Fenton oxidation in batch test. Experimental conditions: H_2O_2 dosage = 120 mg·L^{-1}, [H_2O_2]:[Fe^{2+}] mole ratio = 2:1, T = 293 ± 2 K.

2.4 Optimized reaction condition for Fenton's reaction

In previous studies, the most appropriate reaction conditions for the BDFW were H_2O_2 dosage of 120 mg/L, initial molar ratio of H_2O_2 and ferrous iron of 2:1 and initial pH of 4. The detailed water quality index was measured and compared as presented in Supplementary Table S1. After the Fenton's process, the COD decreased from 156 mg/L to 68 mg/L, and the DOC decreased from 42 mg/L to 23 mg/L. The COD and DOC removal percentages were over 45%. Furthermore, the effluent TP, TSS and colour improved considerably compared to the influent. These indexes can meet the effluent standard of GB 4287-2012. The effluent BOD_5 was much higher than the BDFW, and the BOD_5/COD value also improved to a higher level, which is an

advantage for advanced biochemical treatment. However, the Fenton's reaction had a rare effect on nitrogen removal, which still did not meet the discharge standard.

The Fenton's reaction also had an effect on the SMPs removal. The concentration of protein and polysaccharide in BDFW were 45 mg/L and 35 mg/L, respectively, with corresponding COD levels of 67.5 mg/L and 42 mg/L, respectively (the conversion factors were 1.5 and 1.2, respectively)[12]. Therefore, protein and polysaccharide in BDFW accounted for 66% of the SCOD. After the Fenton's process, protein decreased to approximately 15 mg/L, which is one-third of the influent. Furthermore, polysaccharide also had a removal percentage of 14%, which still contributes significantly to the 36 mg/L COD of the effluent.

2.5 Specific data for MW distribution and UHPLC-QTOF

Molecular Weight Distributions of both BDFW and rapid Fenton oxidation effluent from full-scale plant were analysed. The results are shown in two pdf files. One is entitled Molecular Weight Distribution of BDFW from full-scale plant for BDFW.pdf for BDFW, the other is entitled Molecular Weight Distribution of rapid Fenton oxidation effluent from full-scale plant.pdf for Fenton oxidation effluent.

Water quality of both BDFW and rapid Fenton oxidation effluent from full-scale plant were analysed by UHPLC-QTOF. The results are shown in two excel files. One is entitled Data of full scan of esi - spectra from UHPLC-QTOF of BDFW and Fenton oxidation effluent from full-scale plant.csv, other is entitled Data of full scan of esi + spectra from UHPLC-QTOF of BDFW and rapid Fenton oxidation effluent from full-scale plant.csv

References

1. Lowry, O. H., Rosebrough, N. J., Farr, A. L. & Randall, R. J. Protein measurement with the folin phenol reagent. *The Journal of Biological Chemistry*, **193 (1)**, 265-275 (1951).
2. Frølund, B., Palmgren, R., Keding, K. & Nielsen, P. H. Extraction of extracellular polymers from activated sludge using a cation exchange resin. *Water Research*, **30 (8)**, 1749-1758 (1996).
3. APHA. Standard methods for the examination of water and wastewater, 20th. Washington DC, USA: American Public Health Association/ American Water Works Association/ Water Environmental Federation (1998).
4. Liu, Z. W., Li, W. X., Ma, W. H., Yin, Z. L. & Wu, G. B. Comparison of deep desulfurization methods in alumina production process. *Journal of Central South University*, **22(10)**, 3745-3750 (2015).
5. Canizares, P., Paz, R., Sáez, C. & Rodrigo, M. A. Costs of the electrochemical oxidation of wastewaters: a comparison with ozonation and Fenton oxidation processes. *Journal of Environmental Management,* **90(1),** 410-420 (2009).
6. Cao, G. M., Sheng, M., Niu, W. F., Fei, Y. L. & Li, D. Regeneration and reuse of iron catalyst for Fenton-like reactions. *Journal of Hazardous Materials*, **172(2)**, 1446-1449 (2009).
7. Ben, W., Qiang, Z., Pan, X. & Chen, M. Removal of veterinary antibiotics from sequencing batch reactor (SBR) pretreated swine wastewater by Fenton's reagent. *Water Research*, **43(17)**, 4392-4402 (2009).
8. Kang, Y. W., Cho, M. J. & Hwang, K. Y. Correction of hydrogen peroxide interference on standard chemical oxygen demand test. *Water Research*, **33(5)**, 1247-1251 (1999).
9. Gogate, P.R. and Pandit, A.B. A review of imperative technologies for wastewater treatment I: oxidation technologies at ambient conditions. *Advances in Environmental Research*, **8**, 501-551 (2004).
10. Gallard, H. & De Laat, J. Kinetics of oxidation of chlorobenzenes and phenyl-ureas

by Fe (II)/H_2O_2 and Fe (III)/H_2O_2. Evidence of reduction and oxidation reactions of intermediates by Fe (II) or Fe (III). *Chemosphere*, **42(4)**, 405-413 (2001).

11. Lin, S.H. & Lo, C.C. (1997) Fenton process for treatment of desizing wastewater. *Water Research*, **31**, 2050-2056 (1997).
12. Aquino, S. F. & Stuckey, D. C. Soluble microbial products formation in anaerobic chemostats in the presence of toxic compounds. *Water Research*, **38 (2)**, 255-266 (2004).

Figure captions

Figure S1 Effect of H_2O_2 dosage on SCOD and DOC removal for real BDFW by Fenton oxidation in batch test. Experimental conditions: $[H_2O_2]$:$[Fe^{2+}]$ mole ratio = 5:1, pH_0 = 4.0, T = 293 ± 2 K.

Figure S2 Effect of the initial $[H_2O_2]$:$[Fe^{2+}]$ mole ratio on SCOD and DOC removal for real BDFW by Fenton oxidation in batch test. Experimental conditions: H_2O_2 dosage = 120 $mg \cdot L^{-1}$, pH_0 = 4.0, T = 293 ± 2 K.

Figure S3 Effect of the initial pH on SCOD and DOC removal for real BDFW by Fenton oxidation in batch test. Experimental conditions: H_2O_2 dosage = 120 $mg \cdot L^{-1}$, $[H_2O_2]$:$[Fe^{2+}]$ mole ratio = 2:1, T = 293 ± 2 K.

Table captions

Table S1 Water quality of BDFW and Fenton effluent for real BDFW in batch test

Table S2 Orthogonal array for real BDFW in batch test

Table S3 Reaction conditions and results of orthogonal experiment for real BDFW in batch test
