# Supplementary material for: A Rapid Fenton treatment of bio-treated dyeing and finishing wastewater at second-scale intervals: kinetics by stopped-flow technique and application in a full-scale plant
Source: Sci Rep. 2019 Jul 4;9:9689. doi: 10.1038/s41598-019-45948-9 (PMC6609656; doi:10.1038/s41598-019-45948-9)
Supplement: Supplementary file 2 — Supplementary Info 2 [file 41598_2019_45948_MOESM2_ESM.pdf]

Molecular Weight Distribution of BDFW from full-scale plant
for

A **Rapid Fenton treatment of bio-treated dyeing and finishing wastewater at second-scale intervals: kinetics by stopped-flow technique and application in a full-scale plant**

Yunlu Chen [a], Yunqin Cheng [a], Xiaohong Guan [b], Yan Liu [a,], Jianxin Nie [a], Chenxi Li [a]*

[a] *Department of Environmental Science and Engineering, Fudan University, Shanghai 200433, China*

[b] *College of Environmental Science and Engineering, Tongji University, Shanghai 200092, China*

**Corresponding author. Tel.: +86-21-6564-3894; fax: +86-21-6564-3597;*

Email: liuyan@fudan.edu.cn

===

Operater : CYL
Sequence : 4
Instrument : Instrument 1
Situation : Sample 4
Injection Date : 2015-9-11 10:12:23
Injuction Times:1
Injuction Volumn : 20.000 µl
Acquisition method : D:\STUDENT\CYL\DATA\2015-09-11 2015-09-11 07-56-48\SECmethod-Injection.M
Last Review : 2014-4-26 9:34:25 : HY
Analysis Method : C:\CHEM32\1\METHODS\DEF_LC.M
Last Review : 2015-9-11 11:18:24 : LYX

Additional Information: Peak has been manually integrated

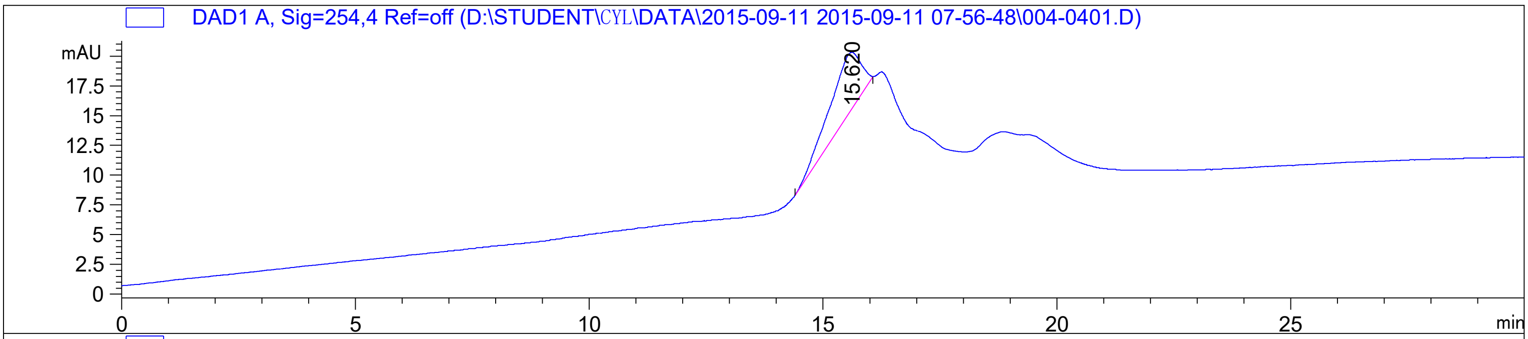

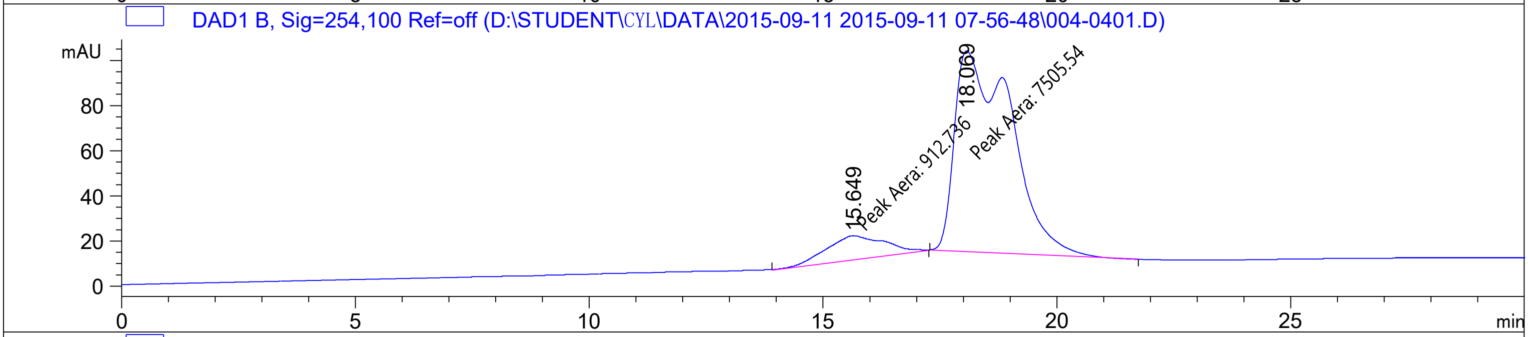

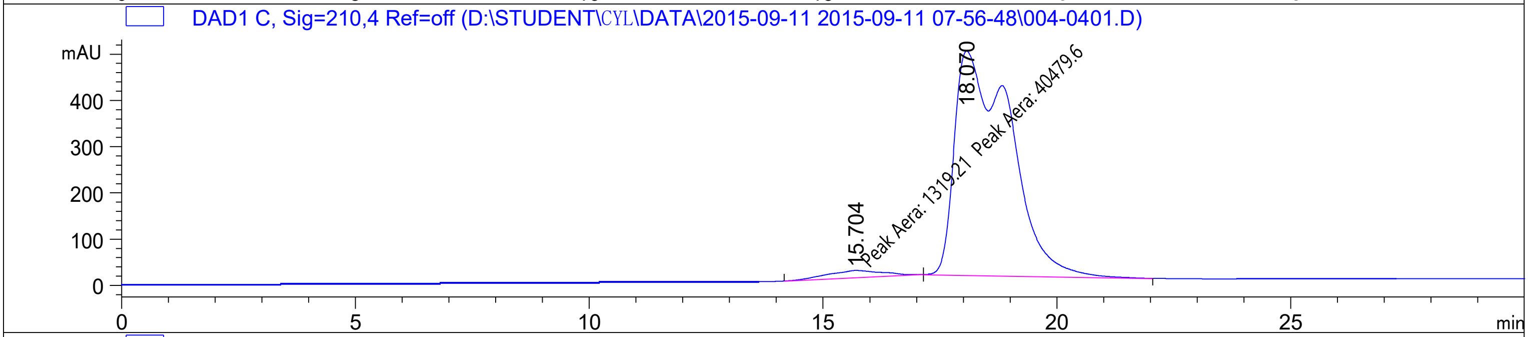

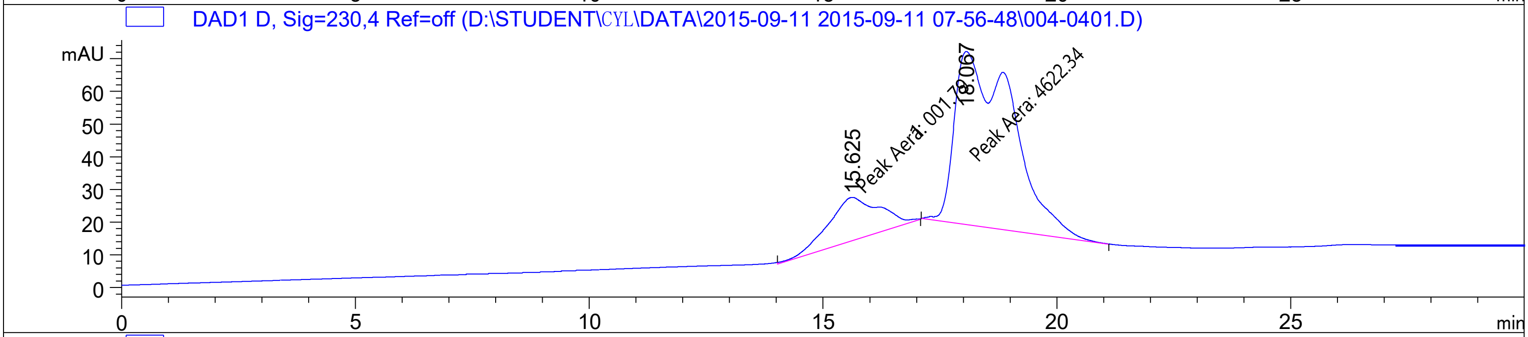

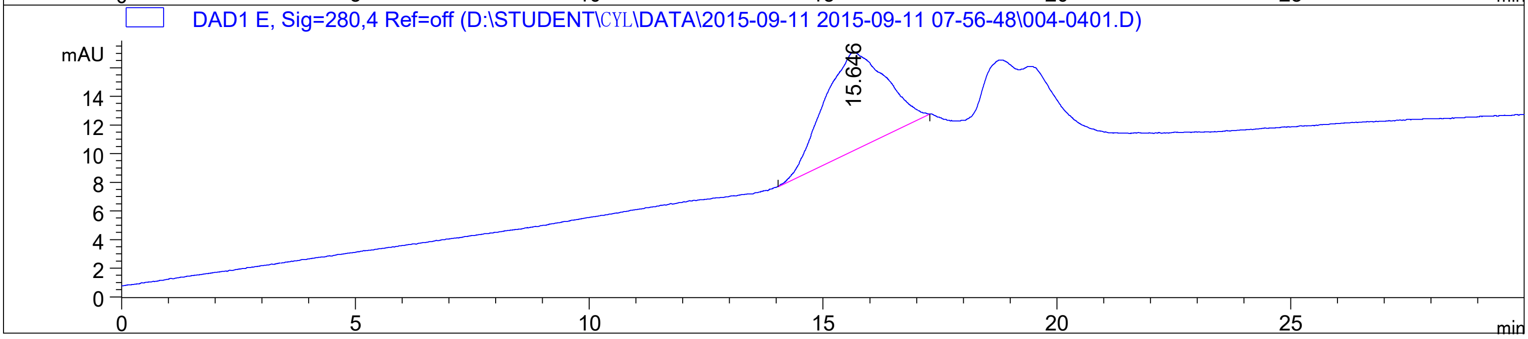

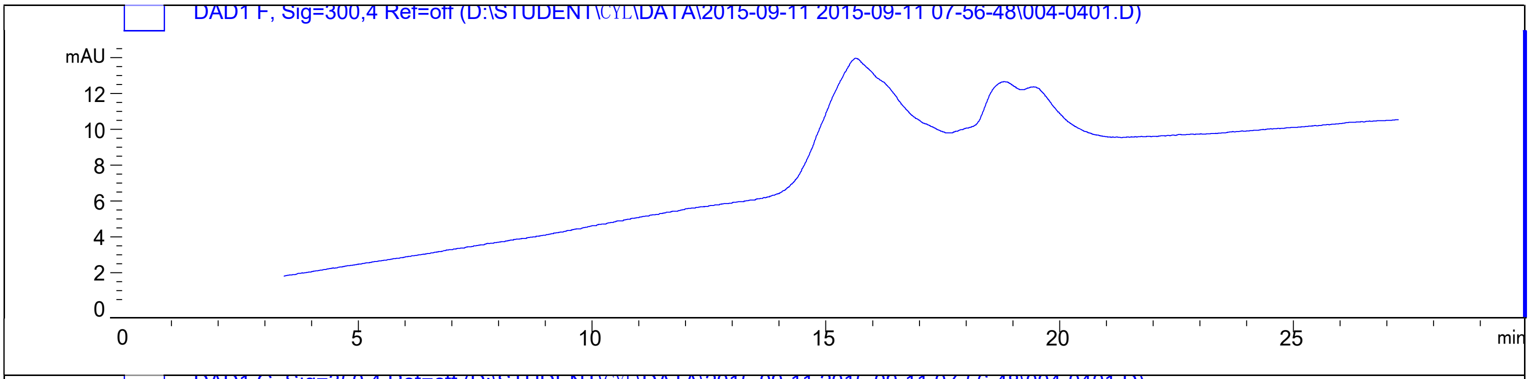

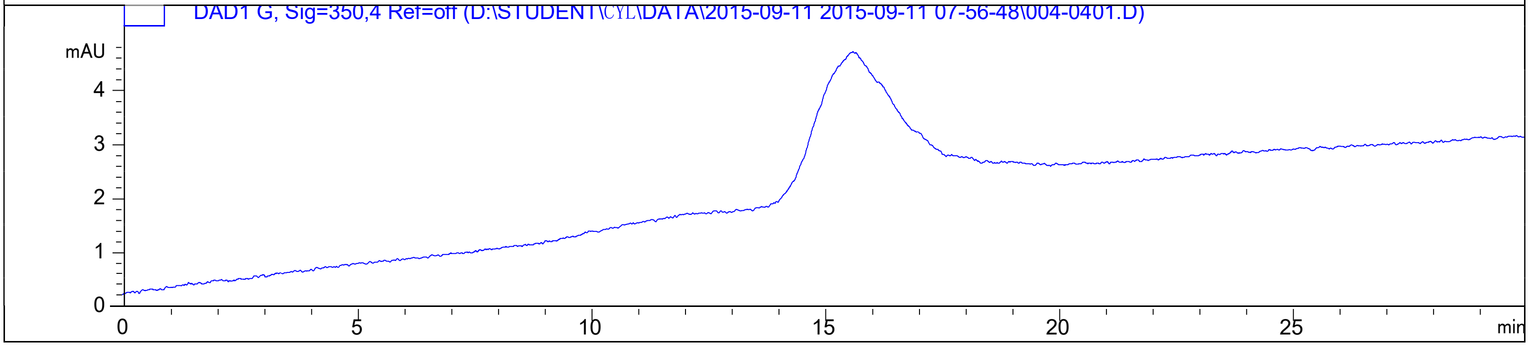

===

Peak Aera Percent Report

===

Sequence : Singal
Multiplication factor: : 1.0000
Dilution factor: : 1.0000
Internal standard using multiplication and dilution factor

Signal 1: DAD1 A, Sig=254,4 Ref=off

Peak #	Retention Time [min]	Type	Peak width [min]	Peak aera [mAU*s]	Peak height [mAU]	Peak aera percentage %
1	15.620	BB	0.6459	231.97055	4.69896	100.0000

Total : 231.97055 4.69896

Signal 2: DAD1 B, Sig=254,100 Ref=off

Peak #	Retention Time [min]	Type	Peak width [min]	Peak aera [mAU*s]	Peak height [mAU]	Peak aera percentage %
1	15.649	MM	1.4274	912.73553	10.65730	10.8423
2	18.069	MM	1.4078	7505.54102	88.85706	89.1577

Total : 8418.27655 99.51437

Signal 3: DAD1 C, Sig=210,4 Ref=off

Peak #	Retention Time [min]	Type	Peak width [min]	Peak aera [mAU*s]	Peak height [mAU]	Peak aera percentage %
1						

	15.704	MM	1.3779	1319.21204	15.95669	3.1561
2	18.070	MM	1.3895	4.04796e4	485.53271	96.8439

Total : 4.17988e4 501.48941

Signal 4: DAD1 D, Sig=230,4 Ref=off

Peak #	Retention Time [min]	Type	Peak width [min]	Peak aera [mAU*s]	Peak height [mAU]	Peak aera percentage %
1	15.625	MM	1.2613	1001.78571	13.23740	17.8123
2	18.067	MM	1.4562	4622.33887	52.90269	82.1877

Total: 5624.12457 66.14009

Signal 5: DAD1 E, Sig=280,4 Ref=off

Peak #	Retention Time [min]	Type	Peak width [min]	Peak aera [mAU*s]	Peak height [mAU]	Peak aera percentage %
1	15.646	BB	1.1000	636.87793	6.89287	100.0000

Total : 636.87793 6.89287

Signal 6: DAD1 F, Sig=300,4 Ref=off

Signal 7: DAD1 G, Sig=350,4 Ref=off

===

*** End of Report ***
