# Supplementary material for: A Rapid Fenton treatment of bio-treated dyeing and finishing wastewater at second-scale intervals: kinetics by stopped-flow technique and application in a full-scale plant
Source: Sci Rep. 2019 Jul 4;9:9689. doi: 10.1038/s41598-019-45948-9 (PMC6609656; doi:10.1038/s41598-019-45948-9)
Supplement: Supplementary file 3 — Supplementary Info 3 [file 41598_2019_45948_MOESM3_ESM.pdf]

Molecular Weight Distribution of rapid Fenton oxidation effluent from full-scale plant for

A Rapid Fenton treatment of bio-treated dyeing and finishing wastewater at second-scale intervals: kinetics by stopped-flow technique and application in a full-scale plant

Yunlu Chen [a], Yunqin Cheng [a], Xiaohong Guan [b], Yan Liu [a,], Jianxin Nie [a], Chenxi Li [a]*

[a] Department of Environmental Science and Engineering, Fudan University, Shanghai 200433, China

[b] College of Environmental Science and Engineering, Tongji University, Shanghai 200092, China

**Corresponding author. Tel.: +86-21-6564-3894; fax: +86-21-6564-3597;*

Email: liuyan@fudan.edu.cn

==

Operater : CYL
Instrument : Instrument 1
Injection Date : 2015-9-11 9:08:57
Injuction Volumn : 20.000 µl
Acquisition method : D:\STUDENT\CYL\DATA\2015-09-11 2015-09-11 07-56-48\SECmethod-Injection.M
Last Review : 2014-4-26 9:34:25 : HY
Analysis Method : C:\CHEM32\1\METHODS\DEF_LC.M
Last Review : 2015-9-11 11:18:24 : LYX

Sequence : 2
Situation : Sample 2
Injuction Times:1

Additional Information: Peak has been manually integrated

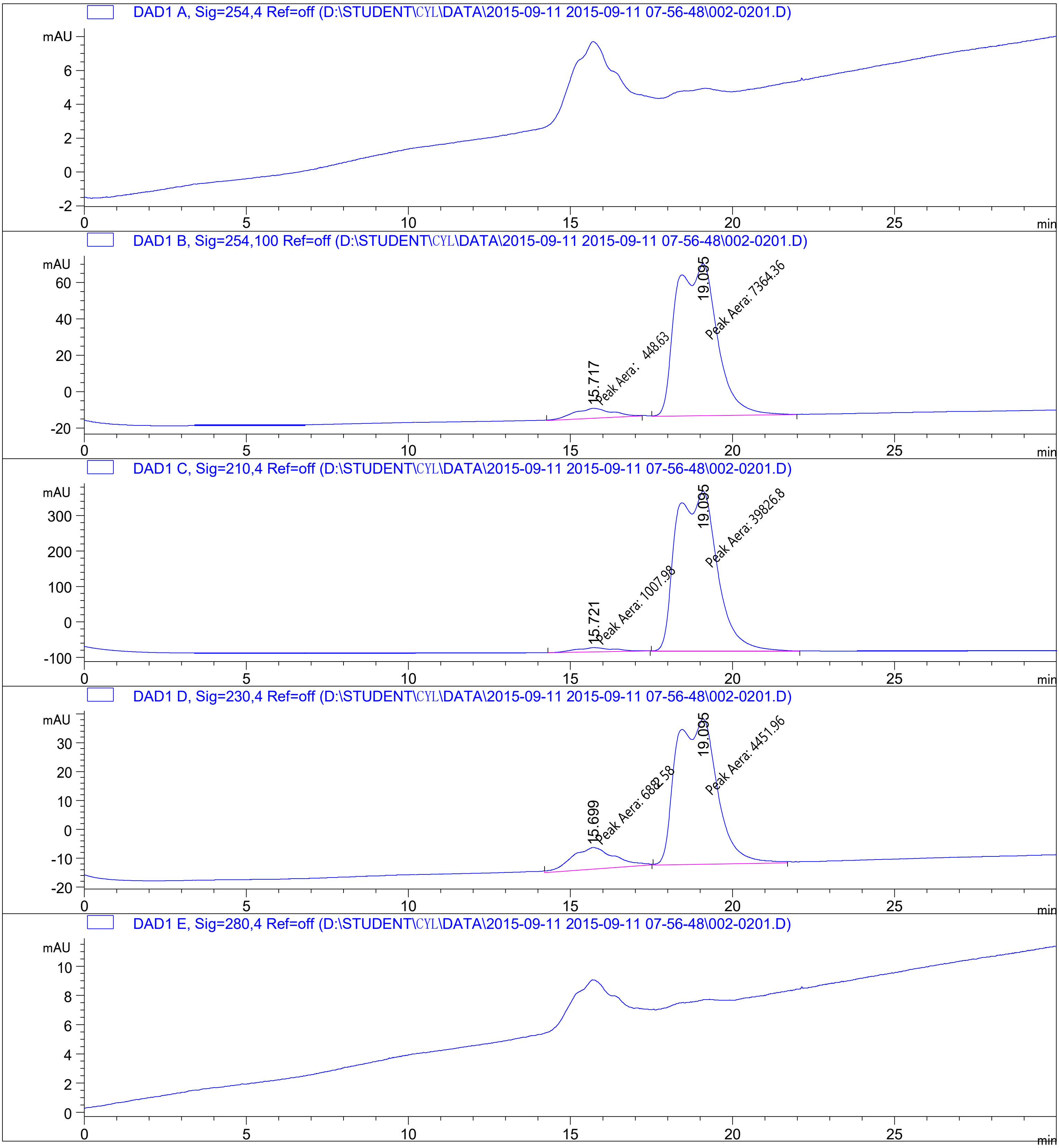

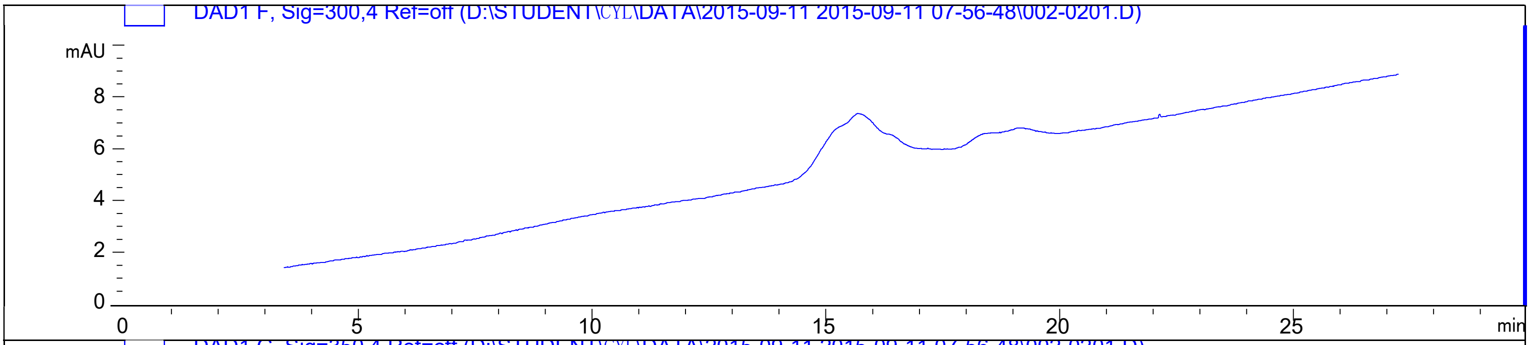

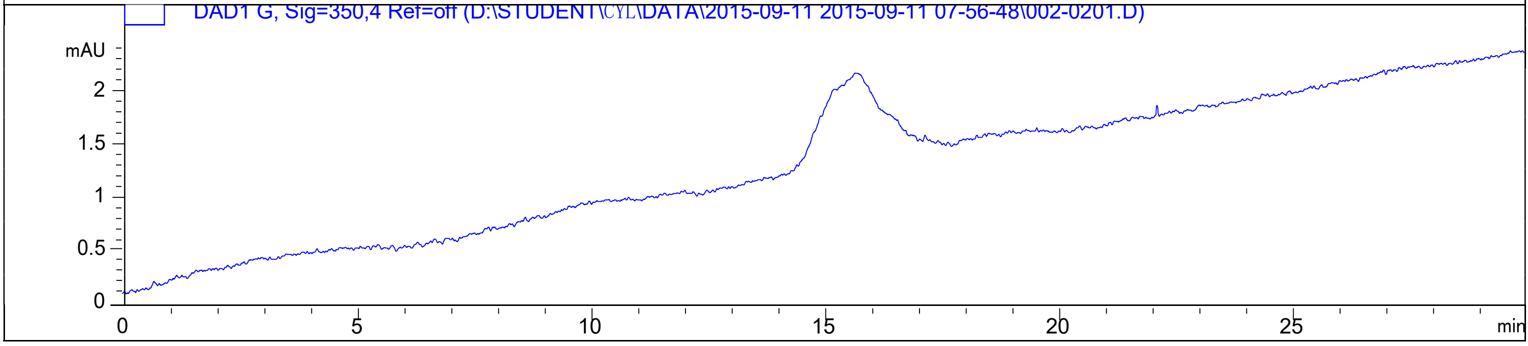

===

Peak Aera Percent Report

===

Sequence : Singal
Multiplication factor: : 1.0000
Dilution factor: : 1.0000
Internal standard using multiplication and dilution factor

Signal 1: DAD1 A, Sig=254,4 Ref=off

Signal 2: DAD1 B, Sig=254,100 Ref=off

Peak #	Retention Time [min]	Type	Peak width [min]	Peak aera [mAU*s]	Peak height [mAU]	Peak aera percentage %
1	15.717	MM	1.3736	448.63040	5.44349	5.7421
2	19.095	MM	1.4699	7364.36133	83.50156	94.2579

Total : 7812.99173 88.94505

信号 3: DAD1 C, Sig=210,4 Ref=off

Peak #	Retention Time [min]	Type	Peak width [min]	Peak aera [mAU*s]	Peak height [mAU]	Peak aera percentage %
1	15.721	MM	1.3741	1007.97668	12.22587	2.4684
2	19.095	MM	1.4726	3.98268e4	450.76682	97.5316

Total : 4.08348e4 462.99269

信号 4: DAD1 D, Sig=230,4 Ref=off

Peak #	Retention Time [min]	Type	Peak width [min]	Peak aera [mAU*s]	Peak height [mAU]	Peak aera percentage %
1	15.699	MM	1.5258	688.25830	7.51815	13.3897
2	19.095	MM	1.4721	4451.96338	50.40315	86.6103
Total :				5140.22168	57.92130	

Signal 5: DAD1 E, Sig=280,4 Ref=off

Signal 6: DAD1 F, Sig=300,4 Ref=off

Signal 7: DAD1 G, Sig=350,4 Ref=off

===

*** End of Report ***
